# Emergence of the Cosmopolitan genotype of dengue virus serotype 2 (DENV2) in Madre de Dios, Peru, 2019

**DOI:** 10.17843/rpmesp.2022.391.10861

**Published:** 2022-03-17

**Authors:** M. Paquita García, Carlos Padilla, Dana Figueroa, Carlos Manrique, César Cabezas

**Affiliations:** 1 Laboratorio Nacional de Referencia de Metaxénicas Virales, Centro Nacional de Salud Pública, Instituto Nacional de Salud, Lima Peru. Laboratorio Nacional de Referencia de Metaxénicas Virales Centro Nacional de Salud Pública Instituto Nacional de Salud Lima Peru; 2 Laboratorio Nacional de Referencia de Biología Molecular y Biotecnología, Centro Nacional de Salud Pública, Instituto Nacional de Salud, Lima Peru. Laboratorio Nacional de Referencia de Biología Molecular y Biotecnología Centro Nacional de Salud Pública Instituto Nacional de Salud Lima Peru; 3 Dirección Ejecutiva de Epidemiología, Dirección Regional de Salud de Madre de Dios, Madre de Dios, Peru. Dirección Ejecutiva de Epidemiología Dirección Regional de Salud de Madre de Dios Madre de Dios Peru; 4 Centro Nacional de Salud Pública, Instituto Nacional de Salud, Lima Peru. Centro Nacional de Salud Pública Instituto Nacional de Salud Lima Peru

*To the editor*. Dengue is a viral disease transmitted by *Aedes aegypti* that is increasingly spreading in tropical and subtropical areas. Up to the epidemiological week 52 (December) of 2019, 3,139,335 cases of dengue were reported in the Americas with deaths (lethality 0.049%); while in Peru during that same epidemiological week, 15,297cases of dengue were reported, of which 76.0% corresponded to the Madre de Dios, Loreto, San Martin, Cajamarca and Tumbes regions. In the Madre de Dios region, 4893 cases were reported, 4.3 times more than in 2018. Of the 13,708 cases in these regions, 3831 (78.3%) showed no alarm signs and 1020 (20.8%) did show alarm signs; 42 (0.86%) were severe and 18 (0.37%) died ^1^.

Dengue virus (DENV) comprises four serotypes, and each serotype is further divided into distinct genotypes. The increase in the incidence of dengue and the severity of the disease have been associated with the change in circulating genotypes as well as viral evolution.

Serotypes DENV1, DENV2, DENV3, and DENV4 have circulated since 2014 in the Madre de Dios region, located in the Peruvian Amazon ^2^. The aim of this study was to show the introduction of a new DENV2 genotype, which does not circulate in the Americas.

During the 2019 dengue outbreak in Madre de Dios, initial diagnosis was made in patients by detecting the NS1 antigen with ELISA. Viral isolation in the C6-36 cell line was successful in 12 samples out of 32 processed. The virus was identified with monoclonal antibodies by indirect immunofluorescence, and simultaneously the samples were analyzed by real-time RT-PCR (qPCR-RT) at the National Referrral Laboratory of Viral Metaxenics of the Instituto Nacional de Salud (INS). All samples were identified as DENV2.

Following the protocol of Santiago *et al*. ^3^, the amplified and purified products were sequenced by the Sanger method at the INS Molecular Biology Laboratory. The obtained sequences were assembled using the SeqScape software to obtain the complete consensus sequence of the E gene. The complete gene sequences were used for phylogenetic analysis using the MEGA X software and the Maximum Likehook algorithm. In addition, the support of each clade was evaluated using the Bootstrap method.

**Figure 1:**
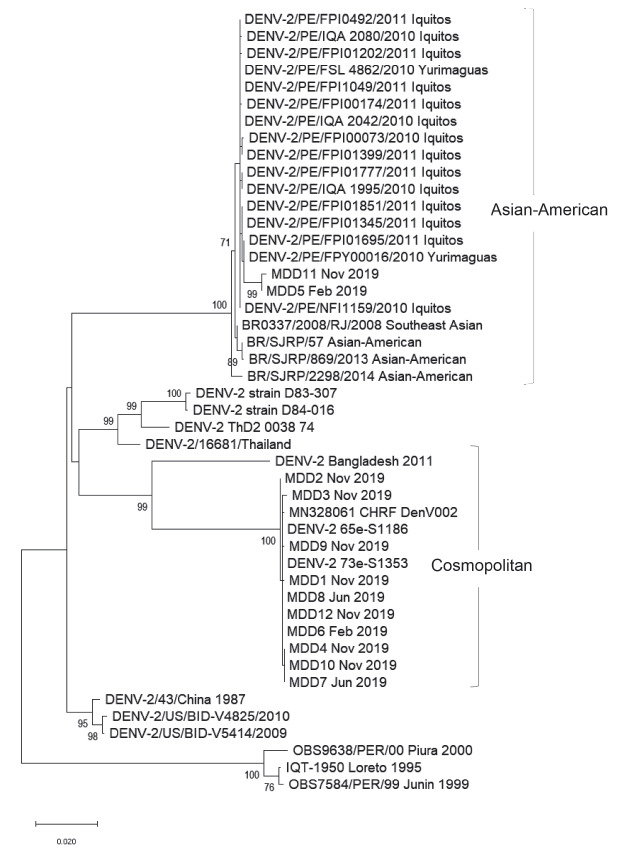
Phylogenetic tree constructed with the Maximum Likehook algorithm showing the homology of DENV2 with the Cosmopolitan genotype from the Madre de Dios-Peru outbreak, 2019. Bootstrap value (>70) is represented at the root of each cluster.

Phylogenetic analysis of twelve evaluated samples groups the sequences into two known DENV2 genotypes; two samples from Madre de Dios cluster in the America/Asia genotype clade that has been circulating in the Loreto region since late 2010 and has been widely distributed since then in Peru. The other ten samples cluster in the Cosmopolitan genotype clade, together with strains from Bangladesh ^4^ and China ^5^, as shown in the phylogenetic tree (Figure 1).

These results demonstrate the introduction of a “new genotype” of DENV2 derived from the Cosmopolitan genotype, highly homologous to the strains reported in Bangladesh. These results were confirmed by the Centers for Disease Control and Prevention, Dengue Branch-Puerto Rico. This Cosmopolitan genotype is circulating in other parts of the world such as India ^4^, Africa ^6^, China ^5^, Indian Ocean and recently (2019) in the French Polynesia ^7^. To our knowledge, this is the first report of an outbreak caused by a Cosmopolitan genotype in the Americas region. The first report of a Cosmopolitan strain detected in the Americas was described in the late 1990s in the Yucatan Peninsula in Mexico, but only one viral isolate was studied and so far, this genotype has not been detected in the Americas region. Determination of the new genotype was made from viral isolates and retrospectively there were limitations to have detailed clinical information.

This finding highlights the need to activate routine genomic surveillance in the laboratory as well as the need for it to include clinical-epidemiological information. It is necessary to conduct genomic surveillance of DENV 2 isolates from other endemic areas of the country to have a broader view of the circulation and to evaluate the route of entry and the spread of this new genotype in the Americas. Additionally, the study should be complemented in order to determine whether or not this new genotype is associated with severe cases of dengue.
