# Supplementary material for: Emergence of the Cosmopolitan genotype of dengue virus serotype 2 (DENV2) in Madre de Dios, Peru, 2019
Source: Rev Peru Med Exp Salud Publica. 2022 Mar 17;39(1):126–8. doi: 10.17843/rpmesp.2022.391.10861 (PMC11397596; doi:10.17843/rpmesp.2022.391.10861)
Supplement: Translation

# Translation: Emergencia del genotipo Cosmopolitan del virus dengue serotipo 2 (DENV2) en Madre de Dios, Perú, 2019

**Authors:** M. Paquita García, Carlos Padilla, Dana Figueroa, Carlos Manrique, César Cabezas

**Affiliations:** 1 Laboratorio Nacional de Referencia de Metaxénicas Virales, Centro Nacional de Salud Pública, Instituto Nacional de Salud, Lima, Perú.; 2 Laboratorio Nacional de Referencia de Biología Molecular y Biotecnología, Centro Nacional de Salud Pública, Instituto Nacional de Salud, Lima, Perú.; 3 Dirección Ejecutiva de Epidemiología, Dirección Regional de Salud de Madre de Dios, Madre de Dios, Perú.; 4 Centro Nacional de Salud Pública, Instituto Nacional de Salud, Lima, Peru.

Sr. Editor. El dengue es una enfermedad viral transmitida por el *Aedes aegypti* que se propaga cada vez más en las zonas tropicales y subtropicales. Hasta la semana epidemiológica 52 (diciembre) de 2019, se reportaron 3 139 335 casos de dengue en América con fallecidos (letalidad 0,049%), mientras que en el Perú en esa misma semana epidemiológica se han reportado 15 297casos de dengue, de los cuales el 76,0% correspondieron a las regiones Madre de Dios, Loreto, San Martín, Cajamarca y Tumbes. En la región de Madre de Dios se han reportado 4893 casos, 4,3 veces más que en 2018. De los 13 708 casos que se dieron en estas regiones, 3831 (78,3%) no mostraron signos de alarma, 1020 (20,8%) mostraron signos de alarma, 42 (0,86%), fueron graves y 18 murieron (0,37%) ^1^.

El virus del dengue (DENV) comprende cuatro serotipos, y cada serotipo se divide a su vez en distintos genotipos. El aumento de la incidencia del dengue y la gravedad de la enfermedad se han asociado al cambio de los genotipos circulantes, así como a la evolución viral. 

En la región de Madre de Dios, ubicada en la Amazonía peruana, los serotipos DENV1, DENV2, DENV3 y DENV4 han circulado desde 2014 ^2^. El objetivo del estudio fue mostrar la introducción de un nuevo genotipo de DENV 2, que no circula en las Américas.

Durante el brote de dengue en Madre de Dios, del año 2019, se hizo el diagnóstico inicial en los pacientes, determinando el antígeno NS1 por ELISA. El aislamiento viral en la línea celular C6-36 fue exitoso en 12 muestras de 32 procesadas. El virus fue identificado con anticuerpos monoclonales por inmunofluorescencia indirecta, y simultáneamente las muestras fueron analizadas por RT-PCR en tiempo real (qPCR-RT) en el Laboratorio de Referencia Nacional de Metaxénicas Virales del Instituto Nacional de Salud (INS). Todas las muestras fueron identificadas como DENV2. 

Siguiendo el protocolo de Santiago *et al*. ^3^, los productos amplificados y purificados se secuenciaron por el método de Sanger en el Laboratorio de Biología Molecular del INS. Las secuencias obtenidas se ensamblaron utilizando el programa informático SeqScape para obtener la secuencia de consenso completa del gen E. Las secuencias completas del gen se utilizaron para realizar un análisis filogenético utilizando el programa informático MEGA X y el algoritmo Maximum Likehook. Además, el soporte de cada clado fue evaluado mediante el método *Bootstrap*.

El análisis filogenético de doce muestras evaluadas agrupa las secuencias en dos genotipos conocidos de DENV2; dos muestras de Madre de Dios se agrupan en el clado del genotipo América/Asia que circula en la región Loreto desde finales del año 2010 y está ampliamente distribuido desde entonces en el Perú. Las otras diez muestras se agrupan en el clado del genotipo Cosmopolitan, junto con cepas de Bangladesh ^4^ y China ^5^, como se muestra en el árbol filogenético (Figura 1).

**Figura 1:** Árbol filogenético construido con el algoritmo Maximum Likehook que muestra la homología del DNEV-2 con el genotipo Cosmopolitan del brote de Madre de Dios-Perú, 2019. El valor Bootstrap (>70) es representado en la raíz de cada clúster.

Estos resultados demuestran la introducción de un «nuevo genotipo» de DENV2 que deriva del genotipo Cosmopolitan, con alta homología con las cepas reportadas en Bangladesh. Estos resultados fueron confirmados por el Centers for Disease Control and Prevention, Dengue Branch-Puerto Rico. Este genotipo Cosmopolitan está circulando en otras partes del mundo como en India ^4^, África ^6^, China ^5^, Océano Índico y recientemente en 2019 en la Polinesia Francesa ^7^. Hasta donde sabemos, este es el primer reporte de un brote causado por un genotipo Cosmopolitan en la región de las Américas. El primer informe de una cepa cosmopolitan detectada en las Américas se describió a finales de los años 90, en la península de Yucatán, en México, pero solo se estudió un aislado viral y hasta ahora no se ha detectado este genotipo en la región de las Américas. La determinación del nuevo genotipo fue hecha a partir de aislamientos virales y retrospectivamente hubo limitaciones para tener información clínica detallada. 

Este hallazgo destaca la necesidad de activar la vigilancia genómica de manera rutinaria en el laboratorio que incluya información clínico-epidemiológica. Siendo necesario realizar la vigilancia genómica de los aislamientos de DENV 2 procedentes de otras zonas endémicas del país para tener una visión más amplia de la circulación, evaluar la ruta de entrada y la dispersión de este nuevo genotipo en las Américas. Adicionalmente, el estudio debe complementarse para determinar si este nuevo genotipo está asociado o no con casos graves de dengue.
